# Association between frequency of breakfast intake before and during pregnancy and developmental delays in children: the Tohoku Medical Megabank Project Birth and Three-Generation Cohort Study

**DOI:** 10.1186/s12937-023-00901-5

**Published:** 2023-12-06

**Authors:** Misato Aizawa, Keiko Murakami, Ippei Takahashi, Hisashi Ohseto, Aoi Noda, Genki Shinoda, Masatsugu Orui, Mami Ishikuro, Taku Obara, Hirotaka Hamada, Noriyuki Iwama, Masatoshi Saito, Junichi Sugawara, Shinichi Kuriyama

**Affiliations:** 1 Graduate School of Medicine, Tohoku University, 2-1 Seiryo-Machi, Aoba-Ku, Sendai, Miyagi 980-8575 Japan; 2 Tohoku Medical Megabank Organization, Tohoku University, 2-1 Seiryo-Machi, Aoba-Ku, Sendai, Miyagi 980-8573 Japan; 3 Department of Pharmaceutical Sciences, Tohoku University Hospital, 1-1 Seiryo-Machi, Aoba-Ku, Sendai, Miyagi 980-8574 Japan; 4 Department of Obstetrics and Gynecology, Tohoku University Hospital, 1-1 Seiryo-Machi, Aoba-Ku, Sendai, Miyagi 980-8574 Japan; 5 Suzuki Memorial Hospital, 3-5-5 Satonomori, Iwanuma, Miyagi 989-2481 Japan; 6 International Research Institute of Disaster Science, Tohoku University, Sendai, Miyagi 980-8572 Japan

**Keywords:** Frequency of breakfast intake, Pregnant, Developmental delays, Japan

## Abstract

**Background:**

Although an association between maternal nutritional intake and developmental delays in children has been demonstrated, the association of the timing of meal intake and development delays remains unclear. We examined the association between breakfast intake frequency before and during pregnancy and developmental delay in children.

**Methods:**

Of the pregnant women who participated in the Tohoku Medical Megabank Project Three-Generation Cohort Study, 7491 answered the required questions and were analyzed. The frequency of breakfast intake from pre- to early pregnancy and from early to mid-pregnancy was classified into four groups: daily, and 5–6, 3–4, and 0–2 times/week. Child developmental delays at age 2 and 3.5 years were assessed using the Ages & Stages Questionnaire, Third Edition. Logistic regression models were constructed to examine the association between breakfast intake frequency in pregnant women and developmental delays in children aged 2 and 3.5 years.

**Results:**

The proportion of pregnant women who had breakfast daily was 78.1% in pre- to early pregnancy, and 82.2% in early to mid-pregnancy. The proportion of children with developmental delays was 14.7% and 13.4% at age 2 and 3.5 years, respectively. Compared with the risk in children of women who had breakfast daily from pre- to early pregnancy, children of women who had breakfast 0–2 times/week had a higher risk of developmental delays at 2 years of age: odds ratio (OR) 1.30, (95% confidence interval [CI], 1.02–1.66). The risk of developmental delays at age 2 years increased in the children of women who had breakfast 0–2 times/week in early to mid- pregnancy: OR 1.75 (95% CI, 1.32–2.32). The risk of developmental delays at age 3.5 years did not increase in the children of women who had breakfast 0–2 times/week from pre- to early and early to mid-pregnancy: OR 1.06 (95% CI, 0.81–1.39 and OR 1.15 (95% CI 0.84–1.57), respectively.

**Conclusion:**

For women with a low frequency of breakfast intake from pre- to mid-pregnancy there was an association with developmental delays in their children at age 2, but not at 3.5 years.

**Supplementary Information:**

The online version contains supplementary material available at 10.1186/s12937-023-00901-5.

## Introduction

Developmental delay is the failure to meet developmental milestones compared to peers [1]. Delays are observed in speech, language, and cognitive development, and also in the ability to adapt to society [2]. Children’s developmental delays result in public health problems with a range of negative consequences, including academic and career difficulties [3], suicide [4] and premature death [5]. The number of children with developmental delays is increasing [6], affecting approximately 10% of children worldwide [7]. Genetic and environmental factors during fetal life may contribute to developmental delays in children [8, 9].

Studies have demonstrated associations between nutrients or foods consumed by pregnant women and developmental delays in the child, including folic acid and multivitamin supplements [10], vegetables, fruit [11], and fish [12], which have been shown to be associated with a reduced risk of developmental delays in the child. The association between dietary patterns such as the Mediterranean and Western diets and developmental delays in children remains unclear [13].

Irregular lifestyle habits of mothers may be associated with developmental delays in their children. Skipping breakfast is a component of irregular lifestyle habits [14], and regular breakfast intake has been associated with the formation of an accurate circadian rhythm [15] and a lower risk of lifestyle-related diseases [16]. Studies have also demonstrated that skipping breakfast during pregnancy is associated with an increased risk of poor diet quality [17], gestational diabetes [18], hypertensive disorders of pregnancy [19], and reduced birth weight [20]. We hypothesized that skipping breakfast would affect nutrient intake and circadian rhythms, and could be associated with developmental delays in children. However, to the best of our knowledge, no studies have examined the association between maternal skipping of breakfast and developmental delays in their children.

In the present study, we examined the association between the frequency of breakfast intake from pre- to mid-pregnancy and developmental delays in children aged 2 and 3.5 years of age. In addition, we investigated the association between changes in the frequency of breakfast intake from pre- to mid-pregnancy and developmental delays in children to evaluate the appropriate period required for breakfast intake.

## Materials and methods

### Study design and population

**Fig. 1:**
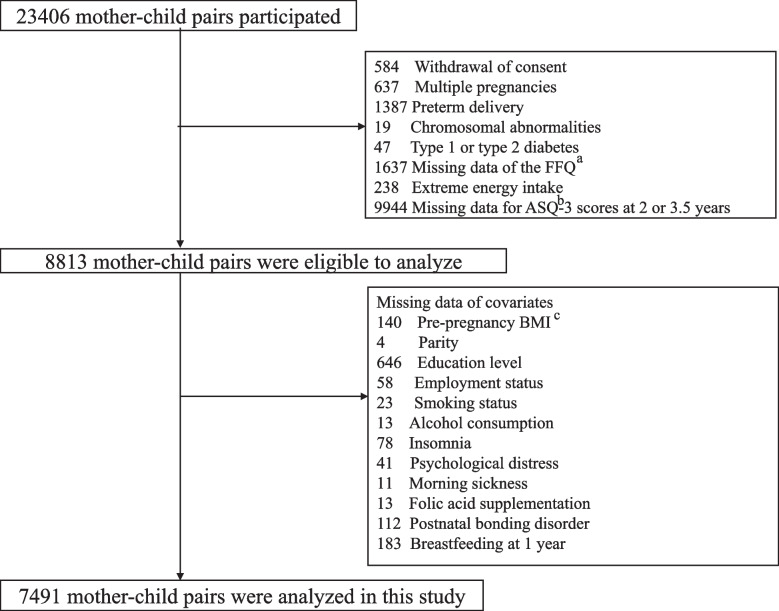
Flowchart of participant inclusion. ^a^FFQ: Food Frequency Questionnaire. ^b^ASQ: Ages and Stages questionnaire. ^c^BMI: body mass index

Data were obtained from the Tohoku Medical Megabank Project Birth and Three-Generation Cohort Study (TMM BirThree Cohort Study), the details of which have been described elsewhere [21]. Pregnant women and their families were recruited between 2013 and 2017 from obstetrics and gynecology clinics or hospitals where deliveries were scheduled. Approximately 50 obstetrics and gynecology clinics and hospitals in Miyagi Prefecture participated in the recruitment process. In total, 23,406 mothers and children were enrolled in this study. Among them, 14,593 mother–child pairs were excluded because of incomplete questionnaires, lack of permission to transcribe medical records, birth defects, or type 1 or type 2 diabetes. Of the remaining 8813 mother–child pairs, 1322 were excluded because of missing values for the analytic variables. The remaining 7491 mother–child pairs were included in this study (Fig. 1).

### Exposure variables

The frequency of breakfast and other food intake was assessed using a 130-item self-administered semiquantitative food frequency questionnaire (FFQ). The FFQs used in this study were made by modifying the FFQ used in the Japan Public Health Center-based Prospective study [22, 23] and were added to the response option “constitutionally unable to eat it” to identify genetic factors. The first FFQ assessed the frequency and quantity of foods and beverages consumed in the preceding year, while the second FFQ assessed those consumed since the administration of the first FFQ. The mean response periods for the first and second FFQ completed by the participants and included in the analysis were 19.3 ± 7·0 weeks and 27·5 ± 5·3 weeks of gestation, respectively. Breakfast intake frequency was assessed using the question, “How often do you eat breakfast?”. The categories of intake frequency in the response items were as follows: less than once a month, 1–3 times a month, 1–2 times a week, 3–4 times a week, 5–6 times a week, and daily. The first three categories were combined into 0–2 times per week because of the small sample size. To examine the association between changes in the frequency of breakfast intake from pre- to mid-pregnancy and developmental delays in the child, we defined those who had breakfast daily or 5–6 times a week as “With breakfast intake” and those who had breakfast 3–4 times a week or 0–2 times a week as “Without breakfast intake [17]”. The four categories were defined as follows: women whose response to the first and second FFQ was “With breakfast intake” (With–With); women whose response to the first FFQ was “With breakfast intake” and whose response to the second FFQ was “Without breakfast intake” (With–Without); women whose response to the first FFQ was “Without breakfast intake” and whose response to the second FFQ was “With breakfast intake” (Without–With); and women whose response to the first and second FFQ was “Without breakfast intake” (Without–Without).

### Outcome variables

The Japanese version of the Ages and Stages Questionnaire (ASQ-3), 3rd Edition was used to assess the child’s development [24, 25]. The ASQ-3 is a comprehensive and reliable screening questionnaire that can be used for children aged 1–66 months. Each questionnaire contained 30 questions divided into five developmental domains: “communication”, “fine motor”, “gross motor”, “problem-solving”, and “personal-social”. Each domain has a set of six items, and each item is scored as 10, 5, or 0, corresponding to’ yes, sometimes, or not yet, respectively. The total score ranged from 0 to 60 for each domain. More than two standard deviations below the mean were the referral cut-offs for each developmental domain, and children were identified as having developmental delays if their scores fell below the referral cut-off in any of the five domains [26, 27].

### Covariates

Based on previous studies [11–13, 18–20], the following covariates were selected: age at delivery, pre-pregnancy body mass index (BMI), educational level, smoking status, alcohol consumption, parity, employment status, folic acid supplement intake, morning sickness, insomnia, psychological distress, postnatal bonding disorder, food intake (cereals, meat, seafood, legumes, vegetables, and fruits), child's sex, and breastfeeding at 1 year. Age at delivery, height, pre-pregnancy weight, and parity were ascertained from participants’ medical records. Age at delivery was categorized as < 24, 25–29, 30–34, and ≥ 35 years. Pre-pregnancy BMI was calculated by dividing the pre-pregnancy weight by the square of height, and was divided into three categories: underweight (< 18.5 kg/m^2^), normal (18.5–24.9 kg/m^2^), and overweight/obesity (≥ 25.0 kg/m^2^). Parity was dichotomized into none and more than one. Data on educational level were obtained one year after birth and classified into three categories: high school or lower, college or special training school, and university or higher. Variables related to smoking status, alcohol consumption, folic acid supplementation during early pregnancy, morning sickness, employment status, psychological distress, and insomnia were obtained using a questionnaire administered during early pregnancy. The woman’s smoking status was divided into four categories: never, quit before pregnancy, quit after pregnancy, and current. Alcohol consumption was divided into three categories: never, former, and current. Folic acid supplement intake was dichotomized as yes or no. Morning sickness was classified into four categories: never, former, and current. Employment status was classified into three categories: employed, not employed, and other. Psychological distress was assessed using the Japanese version of the K6 [28, 29], with psychological distress defined as a score of ≥ 5 [29, 30]. Insomnia was assessed using the Japanese version of the Athens Insomnia Scale, [31] and was defined as a score of ≥ 6 [31, 32]. Postnatal bonding disorder was assessed using a questionnaire at one month postpartum and based on the Japanese version of the Mother-to-Infant Bonding Scale [33]. Postnatal bonding disorder was defined as a score on the Japanese version of the Mother-to-Infant Bonding Scale of ≥ 5 [34]. The intake of each food was calculated as the average intake based on the frequency of intake of cereals, meat, seafood, pulses, vegetables, and fruits from pre-pregnancy to early pregnancy, and early to mid-pregnancy, and was adjusted for energy using the residual method [35]. First, total energy and nutrient intakes were calculated using data from the Standard Tables of Food Composition in Japan (Fifth Revised and Enlarged Edition 2005) [36]. Data on the sex of the children were obtained from medical records. As birth weight is a post-exposure covariate and considered a mediator rather than a covariate, we did not adjust for this covariate. Breastfeeding at 1 year of age was determined using data from the child's 1 year old questionnaire.

### Statistical analysis

Statistical analyses were performed using analysis of variance for continuous variables and the chi-square test for categorical variables, to examine the differences in the characteristics of pregnant women according to breakfast intake frequency. Logistic regression analyses were performed to determine the associations between the frequency of breakfast consumption and developmental delays for each of the five domains of the ASQ-3 at 2 and 3.5 years of age. The odds ratio (OR), 95% confidence interval (CI), and P for trends were calculated and adjusted for covariates. The association between changes in the frequency of breakfast intake and developmental delays in children was assessed in the same way as that used to calculate the ORs and 95% CIs. In addition, because stress during pregnancy is a risk factor for developmental delays [37], sensitivity analyses stratified by insomnia, psychological distress, and employment status were performed.

All the statistical analyses were performed using SAS version 9.4 (SAS Institute Inc., Cary, NC, USA). Statistical significance was set at a *p*-value < 0.05.

## Results

Table 1 shows the characteristics of the pregnant women and children according to their breakfast intake frequency. The mean maternal age at the child’s birth was 31.9 ± 4.6 years. For women who had breakfast daily, the frequency was 78.1% and 82.2% in the pre- to early and early to mid-pregnancy periods, respectively. Compared to women who had breakfast daily, those who skipped breakfast more frequently were younger at delivery, had higher rates of overweight/obesity, and psychological distress. They reported a lower education, that it was their first childbirth, and that they experienced insomnia. Women who skipped breakfast more frequently also had lower energy, cereal, and vegetable intakes. The percentage of children with developmental delays was 14.7% at 2 years of age and 13.4% at 3.5 years of age.

**Table 1:** Characteristics of the participants

	Frequency of breakfast consumption (pre-to early pregnancy)								*P*-value^**1**^
	Everyday (*n* = 5854)		5–6 times/week (*n* = 657)		3–4 times/week (*n* = 430)		0–2 times/week (*n* = 550)		
	n (%) or mean (standard deviation)								
**Mother**									
Age at delivery (years)									
< 25	247	(4.2)	52	(7.9)	41	(9.5)	68	(12.4)	< 0.001
25–29	1393	(23.8)	216	(32.9)	145	(33.7)	191	(34.7)	
30–34	2347	(40.1)	244	(37.1)	158	(36.7)	176	(32.0)	
≥ 35	1867	(31.9)	145	()22.1	86	(20.0)	115	(20.9)	
Pre-pregnancy body mass index (kg/m^2^)									
< 18.5	785	(13.4)	88	(13.4)	66	(15.4)	89	(16.2)	< 0.001
18.5–24.9	4475	(76.4)	496	(75.5)	306	(71.2)	373	(67.8)	
≥ 25.0	594	(10.2)	73	(11.1)	58	(13.4)	88	(16.0)	
Educational level									
Junior high school or high school	1635	(27.9)	205	(31.2)	162	(37.7)	219	(39.8)	< 0.001
Technical college or junior college	2310	(39.5)	251	(38.2)	172	(40.0)	200	(36.4)	
University or graduate school	1909	(32.6)	201	(30.6)	96	(22.3)	131	(23.8)	
Smoking status in early pregnancy									
Never	4024	(68.7)	402	(61.2)	234	(54.4)	265	(48.2)	< 0.001
Quit before pregnancy	1355	(23.2)	164	(25.0)	103	(24.0)	126	(22.9)	
Quit after pregnancy	432	(7.4)	77	(11.8)	77	(17.9)	125	(22.7)	
Smoked during early pregnancy	43	(0.7)	14	(2.1)	16	(3.7)	34	(6.2)	
Alcohol consumption in early pregnancy									
Never	2752	(47.0)	295	(44.9)	182	(42.3)	223	(40.6)	0.009
Former	1899	(32.4)	231	(35.2)	155	(36.1)	220	(40.0)	
Current	1203	(20.6)	131	(19.9)	93	(21.6)	107	(19.5)	
Parity									
≥ 1	3485	(59.5)	214	(32.5)	142	(33.0)	157	(28.6)	< 0.001
0	2369	(40.5)	443	(67.5)	288	(67.0)	393	(71.4)	
Employment status in early pregnancy									
Employed	3478	(59.4)	417	(63.5)	264	(61.4)	341	(62.0)	0.07
Not employed	2220	(37.9)	215	(32.7)	151	(35.1)	191	(34.7)	
Other	156	(2.7)	25	(3.8)	15	(3.5)	18	(3.3)	
With folic acid supplementation	3484	(59.5)	421	(64.1)	271	(63.0)	348	(63.3)	0.03
Morning sickness									
Never	811	(13.9)	104	(15.8)	67	(15.6)	89	(16.2)	0.001
Nausea only	2769	(47.3)	270	(41.1)	197	(45.8)	211	(38.4)	
Vomiting, able to eat	1698	(29.0)	219	(33.3)	119	(27.7)	180	(32.7)	
Vomiting, unable to eat	576	(9.8)	64	(9.7)	47	(10.9)	70	(12.7)	
Insomnia	2072	(35.4)	266	(40.5)	185	(43.0)	246	(44.8)	< 0.001
With psychological distress	1842	(31.5)	232	(35.3)	180	(41.9)	248	(45.1)	< 0.001
With postnatal bonding disorder	823	(14.1)	115	(17.5)	86	(20.0)	108	(19.6)	< 0.001
Energy intake (kcal/day)	1662	± 505.1	1574	± 489.9	1553	± 524.7	1457.8	± 543.3	< 0.001
Cereal intake (g/day)	426.5	± 108.9	415.8	± 109.5	390.8	± 103.2	377.2	± 110.0	< 0.001
Meat intake (g/day)	68.7	± 38.1	76.9	± 37.9	79.2	± 37.5	80.0	± 38.2	< 0.001
Fish intake (g/day)	37.6	± 28.8	35.5	± 24.1	38.2	± 29.0	37.2	± 31.5	0.32
Bean intake (g/day)	62.6	± 61.1	58.5	± 56.0	53.4	± 50.4	59.7	± 58.1	0.007
Seaweed intake (g/day)	6.5	± 5.8	5.6	± 4.5	5.8	± 4.9	5.3	± 4.9	< 0.001
Vegetable intake (g/day)	161.6	± 111.5	139.5	± 79.0	145.6	± 76.0	140.0	± 95.6	< 0.001
Fruit intake (g/day)	148.2	± 128.0	141.7	± 117.1	156.6	± 136.3	155.4	± 143.2	0.16
**Child**									
Birth- weight (g)	3064	± 369.8	3069	± 369.3	3036	± 378.5	3027	± 341.4	0.06
Boy	2963	(50.6)	342	(52.1)	212	(49.3)	298	(54.2)	0.34
Breastfeeding at 1 year	3602	(61.5)	393	(59.8)	253	(58.8)	310	(56.4)	0.08
Developmental delays at 2 years of age	835	(14.3)	94	(14.3)	71	(16.5)	105	(19.1)	0.01
Developmental delays at 3.5 years of age	768	(13.1)	98	(14.9)	60	(14.0)	81	(14.7)	0.45

^1^Compared using the chi-square test for categorical variables and an analysis of variance for continuous variables

The characteristics of the 7491 mother–child pairs that were analyzed, and 15,915 excluded mother–child pairs are shown in Supplementary Table 1. Compared to the study sample overall, women in the analysis sample had a higher age at delivery, a higher level of education and a lower rate of developmental delay in their children. The TMM BirThree Cohort Study protocol was reviewed and approved by the Ethics Committee of the Tohoku University Tohoku Medical Megabank Organization (2013–1-103–1).

Table 2 shows the association between the frequency of breakfast intake and developmental delays in children at 2 years of age. Compared to the values associated with children of women who had breakfast daily from pre- to early pregnancy, the adjusted ORs (95% CIs), of developmental delays in children for women who had breakfast 5–6, 3–4, and 0–2 times/week were 0.95 (0.75–1.21), 1.09 (0.83–1.44), and 1.30 (1.02–1.66), respectively. Compared to the values associated with children of women who had breakfast daily from early to mid-pregnancy, the adjusted ORs (95% CIs), of developmental delays in children for women who had breakfast 5–6, 3–4, and 0–2 times/week were 0.98 (0.77–1.25), 1.22 (0.90–1.65), and 1.75 (1.32–2.32), respectively. The association between the frequency of breakfast intake during pregnancy and each of the five ASQ-3 domains at 2 and 3.5 years of age is shown in Supplementary Tables 2 and 3. The communication domain of children of women who had breakfast less than four times/week before and during pregnancy was associated with an increased risk of delays at the age of 2 years, but in none of the developmental domains at age 3.5 years.

**Table 2:** Association between frequency of breakfast intake during pregnancy and children’s developmental delays at 2 years

	**Frequency of breakfast intake**							***P* for trend**^1^
	**Everyday**	**5–6 times/week**		**3–4 times/week**		**0–2 times/week**		
	**Odds ratios (95% confidence intervals)**							
**Pre- to early pregnancy**								
Case/total (%)	835/5854 (14.3)	94/657 (14.3)		71/430 (16.5)		105/550 (19.1)		
Crude	1.00	1.00	(0.80–1.26)	1.19	(0.91–1.55)	1.42	(1.13–1.78)	0.002
Adjusted^2^	1.00	0.95	(0.75–1.21)	1.09	(0.83–1.44)	1.30	(1.02–1.66)	0.04
**Early to mid-pregnancy**								
Case/total (%)	873/6161 (14.2)	92/630 (14.6)		60/352 (17.1)		80/348 (23.0)		
Crude	1.00	1.04	(0.82–1.31)	1.25	(0.94–1.66)	1.81	(1.39–2.34)	< 0.0001
Adjusted^2^	1.00	0.98	(0.77–1.25)	1.22	(0.90–1.65)	1.75	(1.32–2.32)	0.0004

^1^P for trend was calculated as trends across categories

^2^Multivariable logistic models were adjusted for age at delivery, pre-pregnancy body mass index, parity, employment status, educational level, smoking, alcohol intake, morning sickness, insomnia, psychological distress, postnatal bonding disorder, folic acid, intake of cereal, meat, seafood, beans, vegetables, and fruit, child sex, and breastfeeding at 1 year

Table 3 shows the association between breakfast intake frequency and developmental delays at 3.5 years of age. Compared to the values associated with children of women who had breakfast daily from pre- to early pregnancy, the adjusted ORs (95% CIs), of developmental delays in children for women who had breakfast 5–6 times/week, 3–4 times/week, and 0–2 times/week were 1.13 (0.89–1.43), 0.99 (0.74–1.34), and 1.06 (0.81–1.39), respectively. Compared to the values associated with children of women who had breakfast daily from early to mid-pregnancy, the adjusted ORs (95% CIs), of developmental delays in children for women who had breakfast 5–6, 3–4, and 0–2 times/week were 0.95 (0.74–1.23), 1.04 (0.76–1.44), and 1.15 (0.84–1.57), respectively.

**Table 3:** Association between frequency of breakfast intake during pregnancy and children's developmental delays at 3.5 years

	**Frequency of breakfast intake**							**P for trend**^1^
	**Everyday**	**5–6 times/week**		**3–4 times/week**		**0–2 times/week**		
	**Odds ratios (95% confidence intervals)**							
**Pre- to early pregnancy**								
Case/total (%)	768/5854 (13.1)	98/657 (14.9)		60/430 (14.0)		81/550 (14.7)		
Crude	1.00	1.16	(0.92–1.46)	1.07	(0.81–1.43)	1.14	(0.89–1.47)	0.18
Adjusted^2^	1.00	1.13	(0.89–1.43)	0.99	(0.74–1.34)	1.06	(0.81–1.39)	0.70
**Early to mid-pregnancy**								
Case/total (%)	815/6161 (13.2)	83/630 (13.2)		51/352 (14.5)		58/348 (16.7)		
Crude	1.00	1.00	(0.78–1.27)	1.11	(0.82–1.51)	1.31	(0.98–1.76)	0.08
Adjusted^2^	1.00	0.95	(0.74–1.23)	1.04	(0.76–1.44)	1.15	(0.84–1.57)	0.54

^1^P for trend was calculated as trends across categories

^2^Multivariable logistic models were adjusted for age at delivery, pre-pregnancy body mass index, parity, employment status, educational level, smoking, alcohol intake, morning sickness, insomnia, psychological distress, postnatal bonding disorder, folic acid, intake of cereal, meat, seafood, beans, vegetables, and fruit, child sex, and breastfeeding at 1 year

Table 4 shows the association between changes in breakfast intake frequency and developmental delays in children. Compared to the values associated with children of women who had breakfast daily before and during pregnancy (With–With), the OR (95% CI), for developmental delays at 2 years of age in children of women who only had breakfast pre- to early pregnancy (With–Without) was 1.60 (1.22–2.10). The OR (95% CI), for developmental delays at 2 years of age for children of women who only had breakfast early to mid-pregnancy (Without–With) was 1.06 (0.78–1.43). The OR (95% CI), for developmental delays at 2 years of age in the children of women who did not have breakfast before and during (Without–Without) was 1.16 (0.69–1.97). In a similar comparison with developmental delays at 2 years, the adjusted ORs (95% CIs) of developmental delays at 3.5 years for children were 1.19 (0.89–1.60), 1.01 (0.74–1.40), and 0.62 (0.32–1.21), respectively. Factors in the With–Without group that were associated with children's development at 2 years of age were higher rates of smoking, severe morning sickness, insomnia, psychological distress, and low birth weights.

**Table 4:** Association between frequency changes of breakfast intake from pre- to mid-pregnancy and children’s developmental delays

	**Changes in frequency of breakfast intake**^1^			
	**With -With**	**With -Without**	**Without -With**	**Without -Without**
**Odds ratios (95% confidence intervals)**				
**Children’s developmental delays**				
**Age 2 years**				
Case/total (%)	944/6626 (14.3)	85/389 (21.9)	58/366 (15.9)	18/110 (16.4)
Crude	1.00	1.68 (1.31–2.16)	1.13 (0.85–1.51)	1.18 (0.70–1.96)
Adjusted^2^	1.00	1.60 (1.22–2.10)	1.06 (0.78–1.43)	1.16 (0.69–1.97)
**Age 3.5 years**				
Case/total (%)	880/6626 (13.3)	66/389 (17.0)	51/366 (13.9)	10/110 (9.1)
Crude	1.00	1.33 (1.01–1.76)	1.06 (0.78–1.43)	0.65 (0.34–1.26)
Adjusted^2^	1.00	1.19 (0.89–1.60)	1.01 (0.74–1.40)	0.62 (0.32–1.21)

^1^Changes in the frequency of breakfast intake from pre-pregnancy to mid-pregnancy were defined four categories as follows;

With–With: women whose responses to the first and second FFQs were “with breakfast intake”.

With–Without: women whose response to the first FFQ was “with breakfast intake” and whose response to the second FFQ was “without breakfast intake”.

Without–With: women whose response to the first FFQ was “without breakfast intake” and whose response to the second FFQ was “with breakfast intake”.

Without–Without: women whose answers to the first and second FFQs were “without breakfast intake”.

^2^Multivariable logistic models were adjusted for age at delivery, pre-pregnancy body mass index, parity, employment status, educational level, smoking, alcohol intake, morning sickness, insomnia, psychological distress, postnatal bonding disorder, folic acid, intake of cereal, meat, seafood, beans, vegetables, and fruit, child sex, and breastfeeding at 1 year

Sensitivity analyses stratified by psychological distress, insomnia and employment status showed that the association between breakfast intake frequency and developmental delay in the child was similar to the main results (Supplementary Table 4).

## Discussion

This study showed that less frequent breakfast intake during pre- to mid-pregnancy was associated with an increased risk of developmental delays in children at 2 years of age, but not with developmental delays at 3.5 years of age. To our knowledge, this is the first study to demonstrate an association between breakfast intake frequency in pregnant women and developmental delays in children. In addition, compared to the children of pregnant women who had breakfast during pre- to mid-pregnancy, the children of pregnant women who had breakfast during pre- to early pregnancy, but not during early to mid-pregnancy, had a higher risk of developmental delays at 2 years of age. In contrast, there was no association with developmental delay in children aged 3.5 years of age.

There are several possible reasons why the present study found an association between breakfast frequency in pregnant women and development at 2 years of age. Although different cultures consume different foods at breakfast, a typical Japanese breakfast is rich in soy products, fish, seaweed, vegetables, fruit and green tea [38]. In the present study, mothers who frequently skipped breakfast had a lower intake of energy, cereals, as well as the fish, seaweed and vegetables consumed in a typical Japanese breakfast. However, the association between less frequent breakfast intake in pregnant women and developmental delays in children at 2 years of age remained even after adjusting for these dietary covariates. This result suggests that the mother's irregular lifestyle may influence the child's developmental delays.

At approximately 20 weeks of gestation, the fetus begins to develop an internal clock that is modelled on the mother's circadian rhythm [39]. The regulatory part of the body clock in the brain begins to function at approximately 30 weeks of gestation and matures at approximately 37 weeks of gestation [40]. If the basis of the body clock is not properly established during the fetal period, the supernormal rhythms underlying the body clock will not be properly established [41]. Irregular mealtimes and extreme late nights during pregnancy have been reported to cause disruption of the mother's “biological clock”, which may adversely affect the child's development [42]. A study of pregnant women in Japan showed that low physical activity levels and too little or too much sleep was associated with delayed neurodevelopment of the child [43]. Another study showed that a habit of sleeping past midnight every day and irregular mealtimes were associated with irritable sleep–wake rhythm abnormalities [44]. Thus, irregular mealtimes are detrimental to the physical and mental health of mothers and children, and therefore should be avoided. This study showed that changes in the frequency of breakfast consumption –with breakfast before to early pregnancy and without breakfast in early to mid-pregnancy–were associated with the risk of developmental delays in children aged 2 years. Considering that the biological clock of the fetus begins to form in mid-pregnancy, it seems reasonable that skipping breakfast only during mid-pregnancy affects the formation of the fetal body clock. However, the group that skipped breakfast only in mid-pregnancy was characterized by higher rates of smoking and psychological distress. Therefore, the frequency of breakfast intake is one of the possible pathways and may not fully explain the present association.

Eating breakfast less frequency in pre- to mid-pregnancy was only associated with developmental delays in the communication area in children aged 2 years. In a previous study, pregnant women who had low protein intake in pre- to early-pregnancy and were more likely to skip breakfast had children who were associated with developmental delays of communication, fine motor, and problem-solving skills at 3-years of age [45]. Amino acids, the building blocks of proteins, also act as essential precursors for the synthesis of various molecules such as hormones and neurotransmitters, which may influence neurodevelopment [46]. In this study, pregnant women who ate breakfast less often also had a lower protein intake; however, it is unclear why this was only associated with delayed development in the area of communication.

In the present study, the frequency of breakfast consumption among pregnant women was not associated with developmental delays in children aged 3.5 years. Compared with women whose children had developmental delays at 2 and 3.5 years of age, women whose children had developmental delays at 2 years of age and no developmental delays at 3.5 years of age were more likely to be highly educated, employed, and less likely to have insomnia and stress (Supplementary Table 5). Parents who are better educated and more financially comfortable are likely to invest more in their children’s development [47, 48]. Infant health check-ups are conducted at 18 months of age in Japan, and well-educated parents whose children are assessed as developmentally delayed may have invested more in their children’s development to make up for any developmental delay. Because the period from 0 to 3 years of age is critical for the formation and subsequent maintenance of synapses, inappropriate stimulation during this period can have a significant and lasting negative impact on later development [49]. In addition, interventions between the ages of zero and three years can permanently alter children’s developmental trajectories and protect them from lifestyle-related diseases and depression [50]. In the present study, the frequency with which pregnant women ate breakfast was not associated with developmental delay at the age of 3.5 years. However, increasing the frequency of breakfast consumption in pregnant women to prevent developmental delays at 0–3 years of age may have lifelong benefits for the child.

### Implication

Our findings have implications for the design of effective interventions aimed at preventing developmental delays in children. If the observed association between a low frequency of breakfast intake among pregnant women and child developmental delays is causal, interventions that promote breakfast intake during pregnancy may help prevent child developmental delays. In Japan, the Dietary Guidelines for Pregnant Women have been revised to emphasize the importance of improving dietary habits before pregnancy [51]. Despite the high rate of skipping breakfast among young Japanese women, Dietary Guidelines do not specify breakfast intake. A better understanding of the association between the frequency of breakfast intake before and during pregnancy and child development will help identify at-risk mother–child pairs and implement effective interventions.

### Limitations

This study had several limitations. First, it was conducted in only one of the 47 prefectures in Japan, which limits the generalizability of its findings. Second, the women in this study were older, more educated, and consumed breakfast more frequently than the excluded women did. In addition, the children of the women included in this study had lower rates of developmental delay than those of the children of the excluded women, which could have resulted in a selection bias. Third, data on the frequency of breakfast intake were obtained from participants’ self-reported questionnaires, which could have led to misclassification. Fourth, the ASQ-3 is a screening tool and not a diagnostic tool for developmental delays. However, the reliability and validity of the Japanese version of the ASQ-3 has been confirmed [25]. Fifth, dietary factors other than selected foods may not be adequately adjusted. Although the foods to be adjusted were selected from previous studies [11–13, 18–20], the possibility that other foods may have influenced the association between maternal breakfast frequency and child developmental delay cannot be ruled out. Sixth, despite careful control for known risk factors and unknown potential confounders, we may not have been able to eliminate the effects of residual confounding, which is inevitable in observational studies. However, in the sensitivity analysis stratified by insomnia, psychological distress, and employment status, the same conclusions were reached as in the main analysis.

## Conclusion

A low frequency of breakfast intake before and during pregnancy was associated with developmental delays in the offspring at 2 years of age but not at 3.5 years. Although the mechanisms of this association are not yet fully understood, these findings may provide clues for designing dietary interventions for pregnant women to prevent developmental delays in their children and improve maternal and child health.

### Supplementary Information

**Additional file 1:** **Supplementary Table 1.** Comparison of characteristics of included and excluded participants. **Supplementary Table 2.** Association between frequency of breakfast intake during pregnancy and each of the five domains of ASQ-3 at 2 years of age. **Supplementary Table 3.** Association between frequency of breakfast intake during pregnancy and each of the five domains of ASQ-3 at 3.5 years of age. **Supplementary Table 4.** Analyses stratified by maternal psychological distress, insomnia symptoms and employment status in the association between the frequency of maternal breakfast intake and developmental delays in children at 2 years of age. **Supplementary Table 5.** Characteristics of participants by age of developmental delays.

## Data Availability

The TMM BirThree Cohort Study data that support the findings of this study are not publicly available due to the data containing information that could compromise research participant consent. All inquiries about access to the data should be sent to the TMM.

## Abbreviations

ASQ: Ages and Stages questionnaire
BMI: Body mass index
CI: Confidence interval
FFQ: Food frequency questionnaire
OR: Odds ratio
TMM: Tohoku Medical Megabank Project Birth and Three-Generation Cohort Study (TMM BirThree Cohort Study)
