# Supplementary material for: Association between frequency of breakfast intake before and during pregnancy and developmental delays in children: the Tohoku Medical Megabank Project Birth and Three-Generation Cohort Study
Source: Nutr J. 2023 Dec 6;22:66. doi: 10.1186/s12937-023-00901-5 (PMC10698962; doi:10.1186/s12937-023-00901-5)
Supplement: Supplementary file 1 — Additional file 1: Supplementary Table 1. Comparison of characteristics of included and excluded participants. Supplementary Table 2. Association between frequency of breakfast intake during pregnancy and each of the five domains of ASQ-3 at 2 years of age. Supplementary Table 3. Association between frequency of breakfast intake during pregnancy and each of the five domains of ASQ-3 at 3.5 years of age. Supplementary Table 4. Analyses stratified by maternal psychological distress, insomnia symptoms and employment status in the association between the frequency of maternal breakfast intake and developmental delays in children at 2 years of age. Supplementary Table 5. Characteristics of participants by age of developmental delays. [file 12937_2023_901_MOESM1_ESM.docx]

| **Supplementary table 1. Comparison of characteristics of included and excluded participants** | | | | | |
| --- | --- | --- | --- | --- | --- |
|  | **Participants included**  **(n=7491)** | | **Participants excluded**  **(n=15915)** | | **P value^1^** |
|  | n (%) or mean ±SD | | | |  |
| **Mother** |  |  |  |  |  |
| Age at delivery | 31.9 | ±4.6 | 31.2 | ±5.0 | <0.001 |
| Pre-pregnancy BMI (kg/m^2^) | 21.5 | ±8.1 | 21.2 | ±3.0 | 0.08 |
| Education level |  |  |  |  |  |
| High school graduate or less | 2221 | (30.2) | 5777 | (36.3) | <0.001 |
| Junior or vocational college graduate | 2933 | (39.1) | 6080 | (38.2) |  |
| University graduate or above | 2337 | (31.2) | 4058 | (25.6) |  |
| Smoking status |  |  |  |  |  |
| Never | 4925 | (65.8) | 9023 | (56.7) | <0.001 |
| Quit before pregnancy | 1748 | (23.3) | 3724 | (23.4) |  |
| Quit after pregnancy | 711 | (9.5) | 2657 | (16.7) |  |
| Current | 107 | (1.4) | 511 | (3.1) |  |
| Alcohol consumption |  |  |  |  |  |
| Never | 3452 | (46.1) | 3007 | (18.9) | 0.02 |
| Former | 2505 | (33.4) | 5586 | (35.1) |  |
| Current | 1534 | (20.5) | 7320 | (46.0) |  |
| Parity≥1 | 3998 | (53.4) | 8434 | (53.0) | <0.001 |
| Frequency of breakfast intake |  |  |  |  |  |
| Pre- to early pregnancy |  |  |  |  |  |
| Everyday | 5854 | (78.2) | 11363 | (71.4) | <0.001 |
| 5-6 times/week | 657 | (8.8) | 1718 | (10.8) |  |
| 3-4 times/week | 430 | (5.7) | 1225 | (7.7) |  |
| 0-2 times/week | 550 | (7.3) | 1426 | (10.2) |  |
| Early to mid- pregnancy |  |  |  |  |  |
| Everyday | 6161 | (82.3) | 12206 | (76.7) | <0.001 |
| 5-6 times/week | 630 | (8.4) | 1559 | (9.8) |  |
| 3-4 times/week | 352 | (4.7) | 1034 | (6.5) |  |
| 0-2 times/week | 348 | (4.6) | 1116 | (7.0) |  |
| **Child** |  |  |  |  |  |
| Birth weight | 3060 | ±367.8 | 2963 | ±545.6 | <0.001 |
| Boy | 3815 | (50.9) | 8538 | (54.8) | 0.28 |
| Developmental delays at 2 years of age | 1105 | (14.8) | 2309 | (15.8) | <0.001 |
| Developmental delays at 3.5 years of age | 1007 | (13.4) | 2149 | (14.7) | <0.001 |
| BMI; body mass index | | | | | |
| ^1^Obtained using the Student’s t test for continuous variables and chi-square test for categorical variables, comparing participants who were analyzed and participants who were not. | | | | | |

| **Supplementary table 2. Association between frequency of breakfast intake during pregnancy and each of the five domains of ASQ-3 at 2 years of age** | | | | | | | | | |
| --- | --- | --- | --- | --- | --- | --- | --- | --- | --- |
|  | **Frequency of breakfast intake** | | | | | | | | **P for  trend^1^** |
|  | **Everyday** | | **5-6 times/week** | | **3-4 times/week** | | **0-2 times/week** | |  |
|  | **Odds ratios (95% confidence intervals)** | | | | | | | |  |
| **Pre-to early pregnancy** | |  |  |  |  |  |  |  |  |
| **Communication** | |  |  |  |  |  |  |  |  |
| case/total (%) | 251/5854 (4.3) | | 28/657 (4.3) | | 30/430 (7.0) | | 41 /550 (7.5) | |  |
| Adjusted^2^ | 1.00 | | 1.04 | (0.69-1.57) | 1.61 | (1.06-2.43) | 1.64 | (1.12-2.39) | 0.002 |
| **Gross motor** |  |  |  |  |  |  |  |  |  |
| case/total (%) | 306/5854 (5.2) | | 35/657 (5.3) | | 20/430 (4.7) | | 38/550 (6.9) | |  |
| Adjusted^2^ | 1.00 | | 0.97 | (0.67-1.40) | 0.83 | (0.51-1.33) | 1.33 | (0.92-1.93) | 0.43 |
| **Fine Motor** |  |  |  |  |  |  |  |  |  |
| case/total (%) | 239/5854 (4.1) | | 23/657 (3.5) | | 20/430 (4.7) | | 30/550 (5.5) | |  |
| Adjusted^2^ | 1.00 | | 0.83 | (0.53-1.30) | 1.07 | (0.66-1.74) | 1.30 | (0.86-1.97) | 0.38 |
| **Problem solving** | |  |  |  |  |  |  |  |  |
| case/total (%) | 251/5854 (4.3) | | 28/657 (4.3) | | 22/430 (5.1) | | 26/550 (4.7) | |  |
| Adjusted^2^ | 1.00 | | 0.88 | (0.59-1.32) | 1.04 | (0.65-1.65) | 0.99 | (0.64-1.53) | 0.89 |
| **Personal social** |  |  |  |  |  |  |  |  |  |
| case/total (%) | 286/5854 (4.9) | | 34/657 (5.2) | | 31/430 (7.2) | | 37/550 (6.7) | |  |
| Adjusted^2^ | 1.00 | | 1.05 | (0.72-1.53) | 1.48 | (0.99-2.21) | 1.34 | (0.93-1.98) | 0.05 |
| **Early to mid-pregnancy** | |  |  |  |  |  |  |  |  |
| **Communication** | |  |  |  |  |  |  |  |  |
| case/total (%) | 256/6161 (4.2) | | 35/630 (5.6) | | 25/352 (7.1) | | 34/348 (9.8) | |  |
| Adjusted^2^ | 1.00 | | 1.41 | (0.97-2.06) | 1.82 | (1.16-2.84) | 2.11 | (1.40-3.18) | <0.0001 |
| **Gross motor** |  |  |  | | | | | |  |
| case/total (%) | 321/6161 (5.2) | | 30/630 (4.8) | | 20/352 (5.7) | | 28/348 (8.1) | |  |
| Adjusted^2^ | 1.00 | | 0.87 | (0.59-1.29) | 1.11 | (0.69-1.79) | 1.68 | (1.10-2.59) | 0.08 |
| **Fine Motor** |  |  |  |  |  |  |  |  |  |
| case/total (%) | 247/6161 (4.0) | | 22/630 (3.5) | | 26/352 (7.4) | | 17/348 (4.9) | |  |
| Adjusted^2^ | 1.00 | | 0.86 | (0.54-1.35) | 1.96 | (1.26-3.03) | 1.16 | (0.69-1.97) | 0.12 |
| **Problem solving** | |  |  |  |  |  |  |  |  |
| case/total (%) | 263/6161 (4.3) | | 28/630 (4.4) | | 18/352 (5.1) | | 18/348 (5.2) | |  |
| Adjusted^2^ | 1.00 | | 0.95 | (0.63-1.44) | 1.13 | (0.68-1.88) | 1.15 | (0.69-1.92) | 0.63 |
| **Personal social** |  |  |  |  |  |  |  |  |  |
| case/total (%) | 302/6161 (4.9) | | 36/630 (5.7) | | 18/352 (5.1) | | 32/348 (9.2) | |  |
| Adjusted^2^ | 1.00 | | 1.19 | (0.82-1.73) | 1.12 | (0.68-1.85) | 1.98 | (1.31-2.98) | 0.006 |
| ^1^P for trend was calculated as trends across categories. ^2^ Multivariable logistic models were adjusted for age at delivery, pre-pregnancy body mass index, parity, employment status, educational level, smoking, alcohol intake, morning sickness, insomnia, psychological distress, postnatal bonding disorder, folic acid, intake of cereal, meat, seafood, beans, vegetables, and fruit, child sex, and breastfeeding at 1 year. | | | | | | | | | |

| **Supplementary table 3. Association between frequency of breakfast intake during pregnancy and each of the five domains of ASQ-3 at 3.5 years of age.** | | | | | | | | | |
| --- | --- | --- | --- | --- | --- | --- | --- | --- | --- |
|  | **Frequency of breakfast intake** | | | | | | | | **P for  trend^1^** |
|  | **Everyday** | | **5-6 times/week** | | **3-4 times/week** | | **0-2 times/week** | |  |
|  | **OR (95% CI)** | | | | | | | |  |
| **Pre-to early pregnancy** | |  |  |  |  |  |  |  |  |
| **Communication** | |  |  |  |  |  |  |  |  |
| case/total (%) | 287/5854 (4.9) | | 36/657 (5.5) | | 36/430 (8.4) | | 34/550 (6.2) | |  |
| Adjusted^2^ | 1.00 | | 1.20 | (0.83-1.73) | 1.77 | (1.21-2.60) | 1.25 | (0.84-1.85) | 0.03 |
| **Gross motor** |  |  |  |  |  |  |  |  |  |
| case/total (%) | 233/5854 (3.9) | | 32/657 (4.9) | | 17/430 (4.0) | | 22/550 (4.0) | |  |
| Adjusted^2^ | 1.00 | | 1.16 | (0.79-1.72) | 0.89 | (0.53-1.50) | 0.93 | (0.58-1.49) | 0.76 |
| **Fine Motor** |  |  |  |  |  |  |  |  |  |
| case/total (%) | 316/5854 (5.4) | | 45/657 (6.9) | | 32/430 (7.4) | | 39/550 (7.1) | |  |
| Adjusted^2^ | 1.00 | | 1.15 | (0.82-1.61) | 1.19 | (0.80-1.77) | 1.07 | (0.74-1.56) | 0.55 |
| **Problem solving** | |  |  |  |  |  |  |  |  |
| case/total (%) | 357/5854 (5.9) | | 44/657 (6.5) | | 30/430 (6.8) | | 31/550 (5.4) | |  |
| Adjusted^2^ | 1.00 | | 1.10 | (0.78-1.55) | 1.12 | (0.74-1.69) | 0.90 | (0.60-1.36) | 0.85 |
| **Personal social** |  |  |  |  |  |  |  |  |  |
| case/total (%) | 257/5854 (4.4) | | 30/657 (4.6) | | 23/430 (5.4) | | 26/550 (4.7) | |  |
| Adjusted^2^ | 1.00 | | 0.98 | (0.65-1.46) | 1.07 | (0.67-1.70) | 0.87 | (0.55-1.36) | 0.81 |
| **Early to mid-pregnancy** | |  |  |  |  |  |  |  |  |
| **Communication** | |  |  |  |  |  |  |  |  |
| case/total (%) | 302/6161 (4.9) | | 41/630 (6.5) | | 22/352 (6.3) | | 28/348 (8.1) | |  |
| Adjusted^2^ | 1.00 | | 1.38 | (0.97-1.96) | 1.28 | (0.80-2.03) | 1.43 | (0.93-2.21) | 0.04 |
| **Gross motor** |  |  |  | | | | | |  |
| case/total (%) | 244/6161 (4.0) | | 30/630 (4.8) | | 15/352 (4.3) | | 15/348 (4.3) | |  |
| Adjusted^2^ | 1.00 | | 1.16 | (0.77-1.72) | 1.05 | (0.60-1.81) | 1.03 | (0.59-1.80) | 0.77 |
| **Fine Motor** |  |  |  |  |  |  |  |  |  |
| case/total (%) | 333/6161 (5.4) | | 43/630 (6.8) | | 23/352 (6.5) | | 33/348 (9.5) | |  |
| Adjusted^2^ | 1.00 | | 1.17 | (0.83-1.64) | 1.07 | (0.68-1.69) | 1.43 | (0.94-2.14) | 0.14 |
| **Problem solving** | |  |  |  |  |  |  |  |  |
| case/total (%) | 354/6161 (5.8) | | 40/630 (6.4) | | 24/352 (6.8) | | 24/348 (6.9) | |  |
| Adjusted^2^ | 1.00 | | 1.15 | (0.81-1.63) | 1.23 | (0.79-1.92) | 1.11 | (0.70-1.76) | 0.43 |
| **Personal social** |  |  |  |  |  |  |  |  |  |
| case/total (%) | 265/6161 (4.3) | | 32 /630 (5.1) | | 19/352 (5.4) | | 20/348 (5.8) | |  |
| Adjusted^2^ | 1.00 | | 1.11 | (0.75-1.65) | 1.18 | (0.72-1.94) | 1.01 | (0.61-1.67) | 0.46 |
| ^1^P for trend was calculated as trends across categories. ^2^ Multivariable logistic models were adjusted for age at delivery, pre-pregnancy body mass index, parity, employment status, educational level, smoking, alcohol intake, morning sickness, insomnia, psychological distress, postnatal bonding disorder, folic acid, intake of cereal, meat, seafood, beans, vegetables, and fruit, child sex, and breastfeeding at 1 year. | | | | | | | | | |

| **Supplementary Table 4. Analyses stratified by maternal psychological distress, insomnia symptoms and employment status in the association between the frequency of maternal breakfast intake and developmental delays in children at 2 years of age** | | | | | | | | |
| --- | --- | --- | --- | --- | --- | --- | --- | --- |
|  | **Frequency of breakfast intake** | | | | | | | **P for trend^1^** |
|  | **Everyday** | **5–6 times/week** | | **3–4 times/week** | | **0–2 times/week** | |  |
| **Pre- to ealry pregnancy** | | | | | | | |  |
| Psychological distress | Odds ratios (95% confidence intervals)^2^ | | | | | | |  |
| yes | 1.00 | 0.93 | (0.63-1.37) | 1.28 | (0.86-1.91) | 1.29 | (0.90-1.85) | 0.13 |
| no | 1.00 | 0.96 | (0.71-1.31) | 0.93 | (0.62-1.39) | 1.34 | (0.96-1.87) | 0.28 |
| Insomnia | Odds ratios (95% confidence intervals)^3^ | | | | | | |  |
| yes | 1.00 | 0.77 | (0.52-1.13) | 1.29 | (0.87-1.92) | 1.30 | (0.90-1.88) | 0.13 |
| no | 1.00 | 1.07 | (0.78-1.45) | 0.89 | (0.60-1.32) | 1.31 | (0.94-1.81) | 0.24 |
| Employment status | Odds ratios (95% confidence intervals)^4^ | | | | | | | |
| Employed | 1.00 | 0.98 | (0.72-1.32) | 1.08 | (0.75-1.55) | 1.31 | (0.96-1.79) | 0.14 |
| Not employed | 1.00 | 0.80 | (0.59-1.31) | 1.12 | (0.72-1.75) | 1.29 | (0.88-1.91 | 0.24 |
| **Early to mid-pregnancy** | | | | | | | | |
| Psychological distress | Odds ratios (95% confidence intervals)^2^ | | | | | | | |
| yes | 1.00 | 0.84 | (0.56-1.24) | 1.32 | (0.86-2.02) | 1.76 | (1.19-2.60) | 0.008 |
| no | 1.00 | 1.07 | (0.79-1.46) | 1.09 | (0.71-1.67) | 1.72 | (1.14-2.60) | 0.03 |
| Insomnia | Odds ratios (95% confidence intervals)^3^ | | | | | | |  |
| yes | 1.00 | 1.06 | (0.74-1.53) | 1.37 | (0.87-2.15) | 1.61 | (1.07-2.44) | 0.02 |
| no | 1.00 | 0.9 | (0.65-1.25) | 1.03 | (0.69-1.54) | 1.97 | (1.35-2.88) | 0.01 |
| Employment status | Odds ratios (95% confidence intervals)^4^ | | | | | | | |
| Employed | 1.00 | 1.10 | (0.81-1.49) | 1.21 | (0.81-1.30) | 1.90 | (1.30-2.76) | 0.003 |
| Not employed | 1.00 | 0.81 | (0.54-1.22) | 1.16 | (0.72-1.88) | 1.65 | (1.07-2.52) | 0.005 |
| ^1^P for trend was calculated as trends across categories. | | | | | |  |  |  |
| ^2^ Multivariable logistic models were adjusted for age at delivery, pre-pregnancy body mass index, parity, employment status, educational level, smoking, alcohol intake, morning sickness, insomnia, postnatal bonding disorder, folic acid, intake of cereal, meat, seafood, beans, vegetables, and fruit, child sex, and breastfeeding at 1 year. | | | | | | | | |
| ^3^ Multivariable logistic models were adjusted for age at delivery, pre-pregnancy body mass index, parity, employment status, educational level, smoking, alcohol intake, morning sickness, psychological distress, postnatal bonding disorder, folic acid, intake of cereal, meat, seafood, beans, vegetables, and fruit, child sex, and breastfeeding at 1 year. | | | | | | | | |
| ^4^ Multivariable logistic models were adjusted for age at delivery, pre-pregnancy body mass index, parity, educational level, smoking, alcohol intake, morning sickness, insomnia, psychological distress, postnatal bonding disorder, folic acid, intake of cereal, meat, seafood, beans, vegetables, and fruit, child sex, and breastfeeding at 1 year. | | | | | | | | |

| **Supplementary Table 5. Characteristics of participants by age of developmental delays** | | | | |
| --- | --- | --- | --- | --- |
|  | **With developmental delays age 2, without age 3.5**  **(n=651)** | | **With developmental delays age 2, 3.5**  **(n=494)** | |
|  | n (%) or mean ±SD | | | |
| **Mother** |  |  |  |  |
| Age at delivery | 32.7 | ±4.7 | 32.6 | ±4.5 |
| Pre-pregnancy BMI (kg/m^2^) | 21.4 | ±3.2 | 21.7 | ±4.0 |
| Education level |  |  |  |  |
| High school graduate or less | 177 | (27.2) | 170 | (34.4) |
| Junior or vocational college graduate | 263 | (40.4) | 185 | (37.5) |
| University graduate or above | 211 | (32.4) | 139 | (28.1) |
| Smoking status |  |  |  |  |
| Never | 434 | (66.7) | 328 | (66.4) |
| Quit before pregnancy | 144 | (22.1) | 116 | (23.5) |
| Quit after pregnancy | 64 | (9.8) | 45 | (9.1) |
| Current | 9 | (1.4) | 5 | (1.0) |
| Alcohol consumption |  |  |  |  |
| Never | 307 | (47.2) | 249 | (50.4) |
| Former | 199 | (30.6) | 145 | (29.4) |
| Current | 145 | (22.3) | 100 | (20.2) |
| Parity≥1 | 293 | (45.0) | 223 | (45.1) |
| Employment status in early pregnancy |  |  |  |  |
| Employed | 373 | (57.3) | 267 | (54.1) |
| Not employed | 261 | (40.1) | 216 | (43.7) |
| Other | 17 | (2.6) | 11 | (2.2) |
| Morning sickness |  |  |  |  |
| Never | 91 | (14.0) | 80 | (16.2) |
| Nausea only | 303 | (46.5) | 241 | (48.8) |
| Vomiting, able to eat | 186 | (28.6) | 127 | (25.7) |
| Vomiting, unable to eat | 71 | (10.9) | 46 | (9.3) |
| Insomnia | 247 | (37.9) | 217 | (43.9) |
| With psychological distress | 239 | (36.7) | 221 | (44.7) |
| **Child** |  |  |  |  |
| Birth weight | 3047.1 | ±385.8 | 3052.5 | ±380.4 |
| Boy | 351 | (53.9) | 349 | (70.7) |
| BMI; body mass index | | | | |
| ^1^Obtained using the Student’s t test for continuous variables and chi-square test for categorical variables, comparing participants who were analyzed and participants who were not. | | | | |
